# A Pilot Study of Radiomics Models Combining Multi-Probe and Multi-Modality Images of ^68^Ga-NOTA-PRGD2 and ^18^F-FDG PET/CT for Differentiating Benign and Malignant Pulmonary Space-Occupying Lesions

**DOI:** 10.3389/fonc.2022.877501

**Published:** 2022-06-02

**Authors:** Fei Xie, Kun Zheng, Linwen Liu, Xiaona Jin, Lilan Fu, Zhaohui Zhu

**Affiliations:** ^1^ Beijing Key Laboratory of Molecular Targeted Diagnosis and Therapy in Nuclear Medicine, Department of Nuclear Medicine, Peking Union Medical College Peking Union Medical College (PUMC) Hospital, Chinese Academy of Medical Science and Peking Union Medical College (PUMC), Beijing, China; ^2^ Nanfang PET Center, Nanfang Hospital, Southern Medical University, Guangzhou, China

**Keywords:** ^18^F-FDG, ^68^Ga-NOTA-PRGD2, positron emission tomography computed tomography, pulmonary space-occupying lesions, radiomics

## Abstract

**Background:**

This is a pilot study of radiomics based on ^68^Ga-NOTA-PRGD2 [NOTA-PEG4-E[c(RGDfK)]2)] and ^18^F-FDG PET/CT to (i) evaluate the diagnostic efficacy of radiomics features of ^68^Ga-NOTA-PRGD2 PET in the differential diagnosis of benign and malignant pulmonary space-occupying lesions and (ii) compare the diagnostic efficacy of multi-modality and multi-probe images.

**Methods:**

We utilized a dataset of 48 patients who participated in ^68^Ga-NOTA-PRGD2 PET/CT and ^18^F-FDG PET/CT clinical trials to extract image features and evaluate their diagnostic efficacy in the differentiation of benign and malignant lesions by the Mann-Whitney U test. After feature selection with sequential forward selection, random forest models were developed with tenfold cross-validation. The diagnostic performance of models based on different image features was visualized by receiver operating characteristic (ROC) curves and compared by permutation tests.

**Results:**

Fourteen of the ^68^Ga-NOTA-PRGD2 PET features between benign and malignant pulmonary space-occupying lesions had significant differences (P<0.05, Mann-Whitney U test). Eighteen of the ^68^Ga-NOTA-PRGD2 PET features demonstrated higher AUC values than all CT features in the differential diagnosis of pulmonary lesions. The AUC value (0.908) ​​of the three-modal feature model was significantly higher (P<0.05, permutation test) than those of the single- and dual-modal models.

**Conclusion:**

^68^Ga-NOTA-PRGD2 PET features have better diagnostic capacity than CT features for pulmonary space-occupying lesions. The combination of multi-modality and multi-probe images can improve the diagnostic efficiency of models. Our preliminary clinical hypothesis of using radiomics based on ^68^Ga-NOTA-PRGD2 PET images and multimodal images as a diagnostic tool warrants further validation in a larger multicenter sample size.

## Introduction

^18^F-fluorodeoxyglucose (^18^F-FDG) positron emission tomography/computed tomography (PET/CT), a technology that integrates functional and anatomical imaging, has been applied widely in the discovery, identification and prognostic evaluation of pulmonary space-occupying lesions (1). Cells in abnormal proliferation, infection and inflammation can accumulate the glucose analogue ^18^F-FDG, and malignant lesions usually showed higher ^18^F-FDG uptake than benign lesions. Rise of glucose metabolism can be captured by ^18^F-FDG PET and ^18^F-FDG PET/CT has been proved to be valuable in the diagnosis and differentiation of pulmonary space-occupying lesions (2).

However, ^18^F-FDG is not specific enough as an imaging agent. For one thing, inflammatory cells can show ^18^F-FDG uptake (3, 4), resulting in a high standardized uptake value (SUV) and false positives (5, 6). For another, some malignant tumors with a high degree of differentiation and low metabolism can show low ^18^F-FDG uptake, resulting in false negatives (5, 7).

Integrin receptor imaging has been a promising nuclear medicine imaging method for the diagnosis of lung cancer from pulmonary space-occupying lesions (8). The integrin family plays an important role in a variety of physiological and pathological processes (9). Among them, the integrin receptor αvβ3 is a key molecule involved in the process of tumor angiogenesis, invasion and metastasis (10). Arginine-glycine-aspartic acid (RGD) tripeptide sequences, as one kind of αvβ3 ligand, could reveal the presence of increased angiogenesis in the tumor microenvironment of NSCLC in PET/CT imaging (11). Zheng et al. found that SUVs of ^68^Ga-labeled RGD dimer ^68^Ga-NOTA-PRGD2 PET/CT had better specificity but lower sensitivity than ^18^F-FDG PET/CT in the differential diagnosis of benign and malignant lesions (12).

Since the concept of ‘radiomics’ was proposed in 2012 (13), radiomics analysis based on PET images has been applied to identify lung-occupying lesions before treatment (14).Previous studies have proved the value of radiomics methods based on CT or ^18^F-FDG PET/CT images (15). However, the diagnostic value of radiomics features of ^68^Ga-NOTA-PRGD2 PET images has not been evaluated, and we are curious about how it compare to radiomics methods based on CT or ^18^F-FDG PET/CT. Furthermore, it would be rather valuable to investigate whether multi-modality and multi-probe radiomics method can make a difference in discriminating malignant from benign lesions.

This was the first study performed to extract and evaluate the diagnostic efficacy of radiomics features of ^68^Ga-NOTA-PRGD2 comparing with that of CT or ^18^F-FDG PET/CT in discriminating benign and malignant pulmonary space-occupying lesions. And this is also pilot in establishing and comparing the diagnostic efficacy of radiomics models based on multi-modality and multi-probe images.

## Materials and Methods

### Patients

The study was approved by the Peking Union Medical College Hospital Medical Ethics Committee, and the requirement for informed consent was waived. Forty-eight patients with pulmonary space-occupying lesions in Peking Union Medical College Hospital from 2011 to 2013 were enrolled, and all participated in ^68^Ga-NOTA-PRGD2 PET/CT and ^18^F-FDG PET/CT clinical trials. The pulmonary space-occupying lesions of all included patients were confirmed by pathological diagnosis.

The inclusion criteria were as follows: (a) a single clearly identifiable lung lesion with a volume > 3 cm^3^ on CT and PET images; (b) good image quality; (c) confirmed by a clear pathological diagnosis; and (d) did not receive radiation therapy or chemotherapy prior to PET/CT scanning. The exclusion criteria were as follows: image distortion, motion artifacts, or metal artifacts.

### Image Acquisition and Reconstruction

PET/CT scanning was performed with a Siemens Biograph 128 mCT X PET/CT. All patients were intravenously injected with ^68^Ga-NOTA-PRGD2 of approximately 111 MBq and then underwent CT scanning (120 kV, 50 mAs, pitch 1:1, layer thickness 3 mm, interval 3 mm, matrix 512×512). PET scanning was performed 30 ± 10 minutes after injection (reconstruction method using TrueX, the layer thickness was 3 mm, and the matrix was ​​168×168). Standard routine ^18^F-FDG PET/CT examinations were performed within 1 week. After fasting for at least 6 hours, all patients received low-dose CT and PET scanning 1 hour after an injection of 7.4 MBq/kg ^18^F-FDG.

### Image Segmentation and Feature Extraction

**Figure 1:**
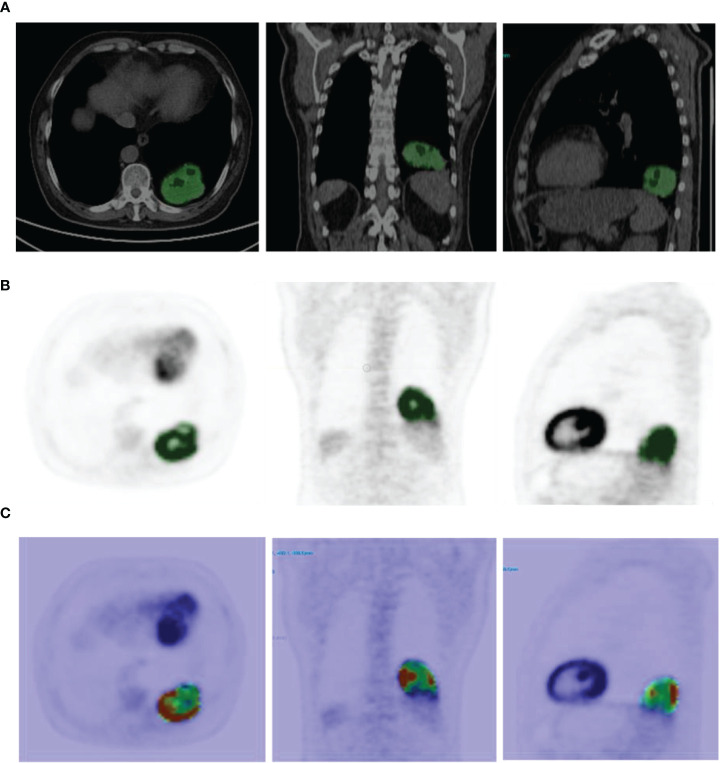
**(A)** Example of CT image segmentation (the green area is the VOI drawn, the same as below). **(B)** Example of ^68^Ga-NOTA-PRGD2 PET image segmentation. **(C)** Example of feature map extracted (GLCM Dissimilarity).

Imaging data were imported into the LIFEx v5.10 (LIFExsoft) platform (16), and an experienced physician manually drew the volume of interest (VOI) layer by layer along the contour of lesions on the PET and CT images. The VOIs (see **Figure 1**) were checked and confirmed by another experienced physician. Basic features, shape features (roundness, density, volume, etc.), and texture features (first-order texture features, gray-level co-occurrence matrix (GLCM) features, gray-level run-length matrix (GLRLM) features, neighborhood gray-level different matrix (NGLMD) features, and gray-level zone length matrix (GLZLM) features) of the VOIs of PET and CT images were extracted (see **Table 1**). Among them, PET features were extracted in the range of SUV values 0-20, and the interval was 64. CT features were extracted in the range of -1500~1000 HU (Hounsfield unit), and the interval was 400. Filtering and smoothing were not used in image processing.

**Table 1:** Image features extracted in our study.

Types	Names of features
SUV conventional features (PET only)	SUV_min_, SUV_mean_, SUV_std_, SUV_max_, SUV_Q1_, SUV_Q2_, SUV_Q3_, SUV_peak sphere 0.5 mL_, SUV_peak sphere 1 mL_, TLG, STLG, MTV, SMTV
HU conventional features (CT only)	HU_min_, HU_mean_, HU_std_, HU_max_, HU_Q1_, HU_Q2_, HU_Q3_, HU_peak sphere 0.5 mL_, HU_peak sphere 1 mL_
Shape features	Volume, Sphericity, Compacity
Textural features	First-order texture features: HISTO Skewness, HISTO Kurtosis, HISTO Excess Kurtosis, HISTO Entropy log10, HISTO Energy
	GLCM features: Homogeneity, Energy, Contrast, Correlation, Entropy log10, Dissimilarity
	GLRLM features: SRE, LRE, LGRE, HGRE, SRLGE, SRHGE, LRLGE, LRHGE, GLNU, RLNU, RP
	NGLDM features: Coarseness, Contrast, Busyness
	GLZLM features: SZE, LZE, LGZE, HGZE, SZLGE, SZHGE, LZLGE, LZHGE, GLNU, ZLNU, ZP

SUV, standard uptake value; SUV_min_, minimum SUV; SUV_mean_, mean SUV; SUV_std_, Standard deviation SUV; SUV_max_, maximum SUV; SUV_Q1_, the first quartile of SUV; SUV_Q2_, the second quartile of SUV; SUV_Q3_, the third quartile; TLG, total lesion glycolysis; STLG, standard total lesion glycolysis; MTV, metabolic tumor volume; SMTV, standard metabolic tumor volume; HU, hounsfield unit; HU_min_, minimum HU; HU_mean_, mean HU; HU_std_, Standard deviation HU; HU_max_, maximum HU; HU_Q1_, the first quartile of HU; HU_Q2_, the second quartile of HU; HU_Q3_, the third quartile of HU; HISTO, Histogram; SRE, Short-Run Emphasis; LRE, Long-Run Emphasis; LGRE, Low Gray-level Run Emphasis; HGRE, High Gray-level Run Emphasis; SRLGE Short-Run Low Gray-level Emphasis; SRHGE, Short-Run High Gray-level Emphasis; LRLGE, Long-Run Low Gray-level Emphasis; RLNU, Run Length Non-Uniformity; RP, Run Percentage; SZE, Short-Zone Emphasis; LZE, Long-Zone Emphasis; LGZE, Low Gray-level Zone Emphasis; HGZE, High Gray-level Zone Emphasis; SZLGE, Short-Zone Low Gray-level Emphasis; SZHGE, Short-Zone High Gray-level Emphasis; LZLGE, Long-Zone Low Gray-level Emphasis; LZHGE, Long-Zone High Gray-level Emphasis; ZLNU, Zone Length Non-Uniformity; ZP, Zone Percentage.

### Data Analysis and Model Establishment

**Figure 2:**
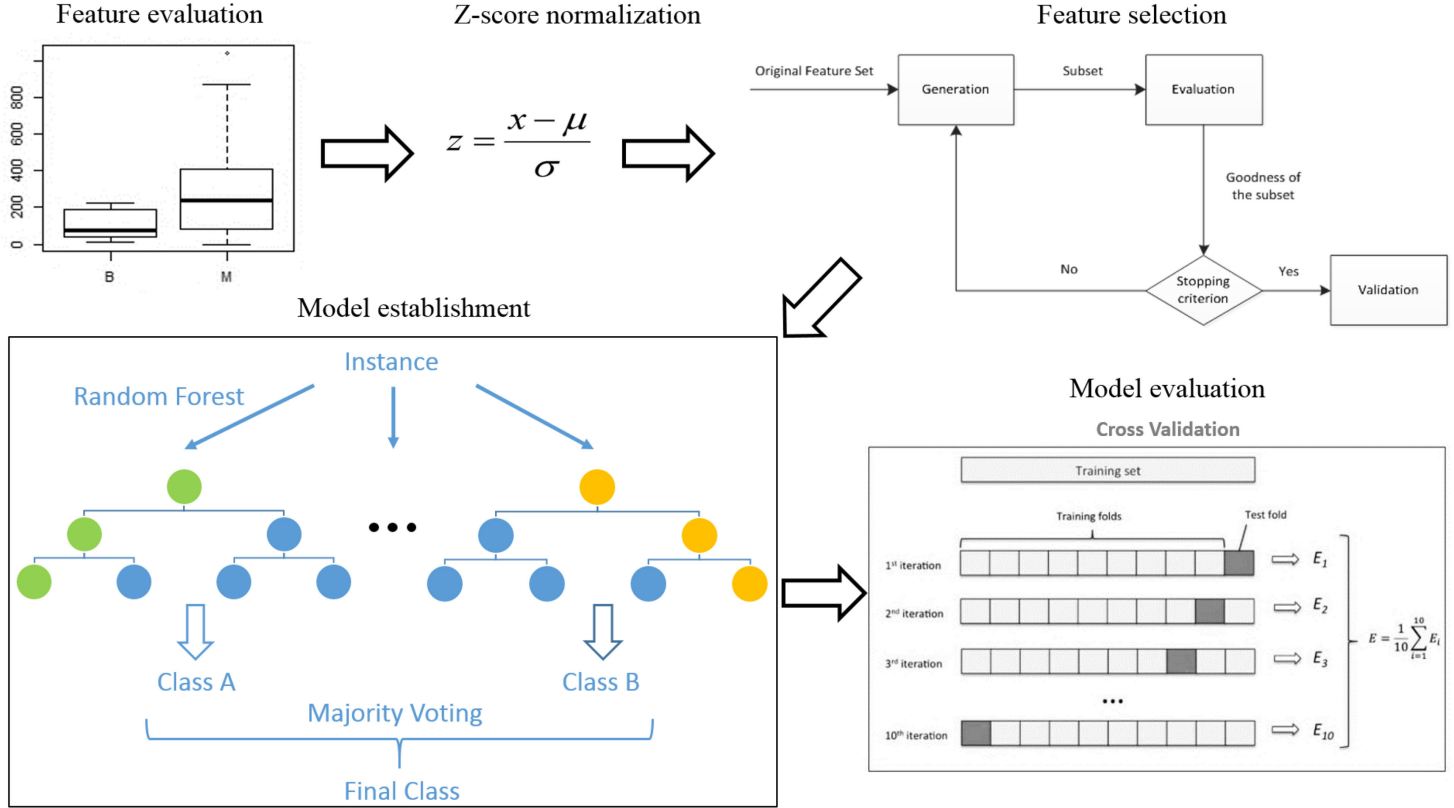
Data analysis and model establishment process.

R 3.5.1 was used to analyze all data. The analysis process was as follows (see **Figure 2**) (1). Feature evaluation: The Mann-Whitney U test was used to compare differences in features between the benign and malignant groups. Correlations were assessed between ^18^F-FDG PET features and ^68^Ga-NOTA-PRGD2 PET features. A single-factor logistic model was established for all radiomics features, and the AUC value of each model was calculated to evaluate the diagnostic efficacy of each feature. (2) Data preprocessing: To avoid some features overpowering others, each feature was standardized (Z-score normalization). (3) Model establishment: This study used the random forest algorithm for modeling. CT features, ^18^F-FDG PET features, ^68^Ga-NOTA-PRGD2 PET features, CT & ^18^F-FDG PET features, CT & ^68^Ga-NOTA-PRGD2 PET features, ^18^F-FDG PET & ^68^Ga-NOTA-PRGD2 PET features, and a combination of all features were used to establish random forest models to predict benign and malignant lesions. The sequence forward selection algorithm was used in the feature selection process. The average AUC value, accuracy, sensitivity, and specificity of the models were evaluated by 1000 times 10-fold cross-validation. The permutation test was used to evaluate the significance of the difference in the mean AUCs of different models.

## Results

### Patient Characteristics

A total of 48 patients were included in this study, of which 23 (47.9%) were men and 25 (52.1%) were women, with an average age of 55.0 years (24~78 years). Among them, 36 cases (75%) were malignant, and 12 cases (25%) were benign. The pathological types were 22 cases of adenocarcinoma, 8 cases of squamous cell carcinoma, 2 cases of malignant mesothelioma, 2 cases of lymphoma, 2 cases of metastasis, 9 cases of chronic inflammation, 1 case of thymoma (AB type), 1 case of hemangioma, and 1 case of epithelioid hemangioendothelioma (see **Table 2**).

**Table 2:** Basic information and characteristics of the patients.

Demographic or Clinical Characteristics	No. of Patients
No. of patients	48
Sex	
Male	23 (47.9%)
Female	25 (52.1%)
Diagnosis	
Malignant	36 (75.0%)
Adenocarcinoma	22 (45.8%)
Squamous cell carcinoma	8 (16.7%)
Malignant mesothelioma	2 (4.2%)
Lymphoma	2 (4.2%)
Metastasis	2 (4.2%)
Benign	12 (25.0%)
Chronic inflammation	9 (18.8%)
Thymoma (AB type)	1 (2.1%)
Hemangioma	1 (2.1%)
Epithelioid hemangioendothelioma	1 (2.1%)
Age (y)	Mean (Range)
	55.0 (24~78)

### Feature Evaluation

**Figure 3:**
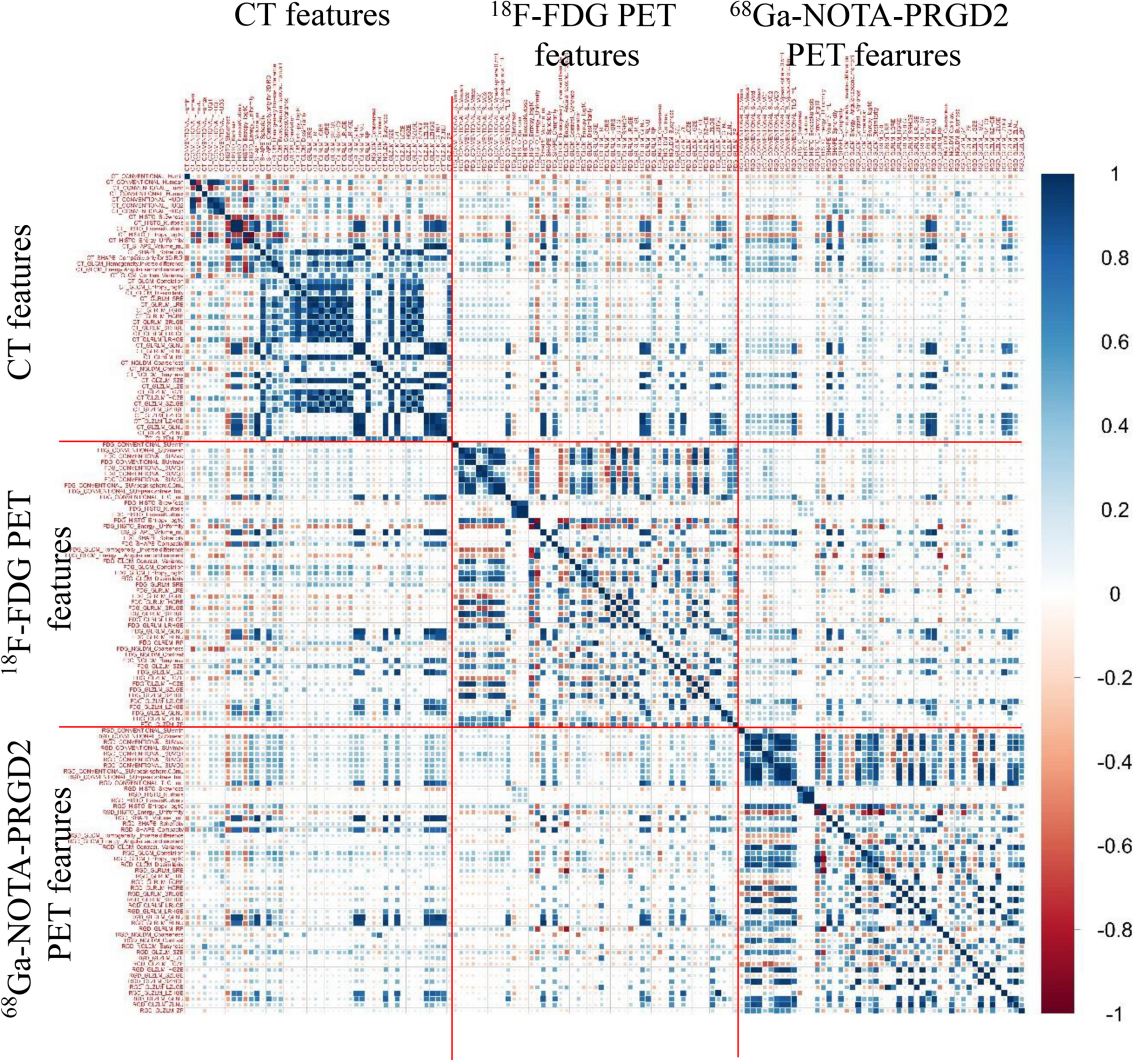
Image feature correlation coefficient map of CT, ^18^F-FDG PET and ^68^Ga-NOTA-PRGD2 PET.

The predictive value (AUC values) of features for benign and malignant lung lesions and the comparison of features between the two groups are shown in **Supplementary Table 1**. Fourteen of the ^68^Ga-NOTA-PRGD2 PET features between benign and malignant pulmonary space-occupying lesions had significant differences (P<0.05, Mann-Whitney U test). The ^18^F-FDG PET feature with the best predictive effect was GLZLM SZLGE with an AUC value of 0.794, the ^68^Ga-NOTA-PRGD2 PET feature with the best predictive effect was GLCM homogeneity with an AUC value of 0.788, and the CT feature with the best predictive effect was HU_max_ with an AUC value of 0.660. Eighteen of the ^68^Ga-NOTA-PRGD2 PET features demonstrated higher AUC values than all CT features in the differential diagnosis of pulmonary lesions. The image feature correlation coefficient map (**Figure 3**) showed that CT, ^18^F-FDG PET and ^68^Ga-NOTA-PRGD2 PET images had certain correlations, but these correlations were lower than the correlations of features within certain modalities.

### Comparison of the Models for Differentiating Benign and Malignant Lesions

**Figure 4:**
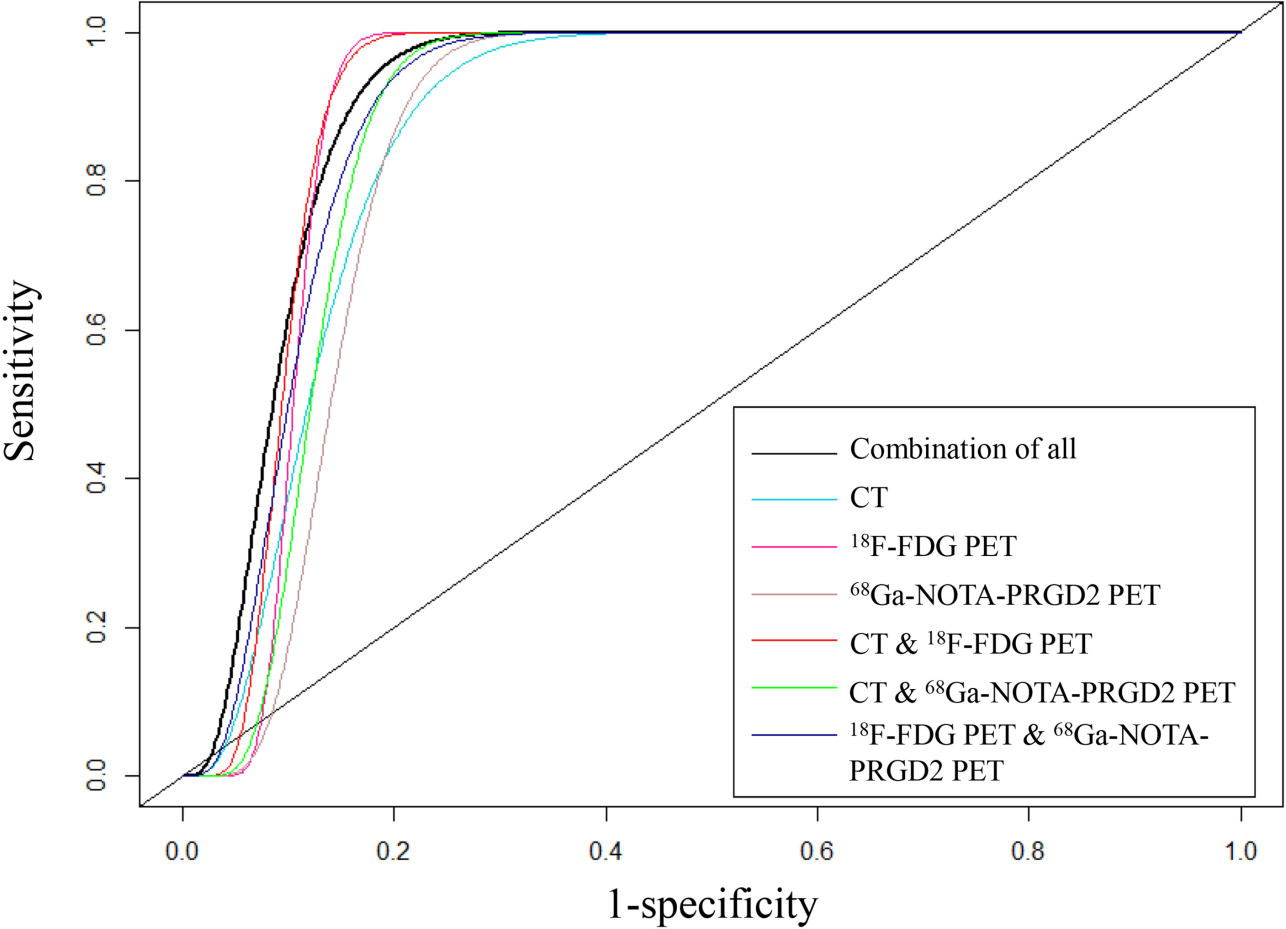
Smoothed ROC curves of all models.

The features included in each model and the AUC, accuracy, sensitivity, specificity and significance test results of these models are shown in **Tables 3**, **4**. The AUC values ​​of the benign and malignant identification models constructed based on CT features, ^18^F-FDG PET features, ^68^Ga-NOTA-PRGD2 PET features, CT & ^18^F-FDG PET features, CT & ^68^Ga-NOTA-PRGD2 PET features, ^18^F-FDG PET & ^68^Ga-NOTA-PRGD2 PET features, and the combination of all were 0.870, 0.895, 0.853, 0.903, 0.876, 0.892 and 0.908; the accuracy rates were 0.770, 0.787, 0.758, 0.805, 0.802, 0.811 and 0.828; the sensitivities were 0.937, 0.932, 0.915, 0.902, 0.943, 0.924 and 0.948; and the specificities were 0.267, 0.351, 0.285, 0.519, 0.378, 0.473, 0.467, respectively. The three-modal image model had a higher AUC than the dual-modal image models in all cases (P<0.05), and the dual-modal image models had a higher AUC than the single-modal image models as its component in most cases (P<0.05). The ^18^F-FDG PET model had a higher AUC than the ^68^Ga-NOTA-PRGD2 PET feature model (P<0.05). The smoothed receiver operating characteristic (ROC) curves of all models are shown in **Figure 4**.

**Table 3:** Feature composition and evaluation results of models for the differentiation of benign and malignant lesions.

Modality	Features included in the model	AUC	Accuracy	Sensitivity	Specificity
CT	HU_max_, GLRLM GLNU, HISTO Skewness, Compacity	0.870 ± 0.048	0.770 ± 0.028	0.937 ± 0.024	0.267 ± 0.091
^18^F-FDG PET	GLZLM SZLGE, HISTO Entropy log10, GLZLM LGZE, Sphericity	0.895 ± 0.038	0.787 ± 0.021	0.932 ± 0.022	0.351 ± 0.058
^68^Ga-NOTA-PRGD2 PET	GLCM Homogeneity, GLRLM LRE	0.853 ± 0.047	0.758 ± 0.024	0.915 ± 0.021	0.285 ± 0.076
CT & ^18^F-FDG PET	^18^F-FDG PET GLZLM SZLGE, CT HU_max_, ^18^F-FDG PET GLZLM LGZE	0.903 ± 0.040	0.805 ± 0.023	0.902 ± 0.019	0.519 ± 0.077
CT & ^68^Ga-NOTA-PRGD2 PET	^68^Ga-NOTA-PRGD2 PET GLCM Homogeneity, CT GLCM Dissimilarity, CT HU_min_, CT Sphericity	0.876 ± 0.044	0.802 ± 0.030	0.943 ± 0.030	0.378 ± 0.074
^18^F-FDG PET & ^68^Ga-NOTA-PRGD2 PET	^18^F-FDG PET GLZLM SZLGE, ^68^Ga-NOTA-PRGD2 PET GLCM Contrast, ^18^F-FDG PET SUVQ2, ^18^F-FDG PET Sphericity	0.892 ± 0.043	0.811 ± 0.021	0.924 ± 0.022	0.473 ± 0.061
CT & ^18^F-FDG PET & ^68^Ga-NOTA-PRGD2 PET	^18^F-FDG PET GLZLM SZLGE, ^68^Ga-NOTA-PRGD2 PET GLCM Contrast, CT GLCM Dissimilarity, CT HU_min_, CT SHAPE Sphericity	0.908 ± 0.041	0.828 ± 0.019	0.948 ± 0.016	0.467 ± 0.070

Data presented as means ± standard deviations.

**Table 4:** Significance test of differences in AUC values of different models.

Modality 1	Modality 2	P values of AUC between modality 1 and modality 2
CT, ^18^F-FDG PET & ^68^Ga-NOTA-PRGD2 PET	CT	<0.001***
	^18^F-FDG PET	<0.001***
	^68^Ga-NOTA-PRGD2 PET	<0.001***
	CT & ^18^F-FDG PET	0.003***
	CT & ^68^Ga-NOTA-PRGD2 PET	<0.001***
	^18^F-FDG PET & ^68^Ga-NOTA-PRGD2 PET	<0.001***
CT & ^18^F-FDG PET	CT	<0.001***
	^18^F-FDG PET	<0.001***
CT & ^68^Ga-NOTA-PRGD2 PET	CT	0.003***
	^68^Ga-NOTA-PRGD2 PET	<0.001***
^18^F-FDG PET & ^68^Ga-NOTA-PRGD2 PET	^18^F-FDG PET	0.108
	^68^Ga-NOTA-PRGD2 PET	<0.001***
^18^F-FDG PET	^68^Ga-NOTA-PRGD2 PET	<0.001***
CT & ^18^F-FDG PET	CT & ^68^Ga-NOTA-PRGD2 PET	<0.001***

***, P<0.01.

## Discussion

Lung cancer is the most common type of malignant lung space-occupying lesion and the main cause of cancer mortality (17). The early diagnosis and treatment of lung cancer from all forms of lung space-occupying lesions have been crucial for the success of surgical resection and extension of survival time (18, 19).

At present, there have been a number of studies on ^18^F-FDG PET/CT radiomics to assist in the differentiation of pulmonary space-occupying lesions (20–25), indicating the excellent value of radiomics in nuclear medicine imaging. It was generally considered that the ^18^F-FDG PET & CT combined model performed better than the PET feature model or CT feature model, and the PET feature model was better than the CT feature model (26). Zhang et al. (27) showed that the AUC of the ^18^F-FDG PET & CT model was higher than that of the CT model (P = 0.018), and the AUC of the ^18^F-FDG PET model was higher than that of the CT model (0.874 *vs*. 0.820) but without a statistically significant difierence. Kang et al. reported that the AUC of the ^18^F-FDG PET model was higher than that of the CT model (0.88 *vs*. 0.74) (22). ^68^Ga-NOTA-PRGD2 PET/CT, an imaging method with dimeric RGD peptide-binding integrin receptors, showed a similar sensitivity and higher specificity than ^18^F-FDG PET/CT in the detection and differentiation of lung lesions (12). However, to the best of our knowledge, there have been no radiomics studies on integrin receptor imaging in lung lesions.

In our study, a variety of intensity, shape and texture features were extracted from the images of 48 patients who underwent ^68^Ga-NOTA-PRGD2 PET/CT and ^18^F-FDG PET/CT scans, and these features were analyzed separately to predict benign and malignant pulmonary lesions. The results showed that ^68^Ga-NOTA-PRGD2 PET and ^18^F-FDG PET features have similar diagnostic capacity for pulmonary space-occupying lesions. ^68^Ga-NOTA-PRGD2 PET image features have better diagnostic capacity than CT, and 18 of the ^68^Ga-NOTA-PRGD2 PET features demonstrated higher AUC values and better diagnostic capacity than all CT features in the differential diagnosis of pulmonary lesions. The radiomics features of the three imaging methods had certain potential predictive value for benign and malignant lung lesions, but the AUC values did not exceed 0.80, which indicated that these features had certain limitations as predictors of benign and malignant lesions alone.

Among the ^18^F-FDG PET features, a total of 11 features had significant differences (P<0.05) between the benign and malignant groups. Among the ^68^Ga-NOTA-PRGD2 PET features, a total of 14 features were significantly different (P<0.05) between the two groups. There were no significant differences (P<0.05) in CT features between the two groups. This finding shows that the features of benign and malignant pulmonary space-occupying lesions on ^18^F-FDG PET or ^68^Ga-NOTA-PRGD2 PET images had certain differences. Since image features are affected by multiple factors (28, 29), such as scanning parameters, reconstruction methods and quantification methods, this difference needs to be further verified. The image feature correlation coefficient map showed that CT, ^18^F-FDG PET and ^68^Ga-NOTA-PRGD2 PET images had certain correlations, but these correlations were lower than the correlations of features within certain imaging methods, suggesting that the information extracted from different sequences could be complementary.

This study further used the sequence forward selection algorithm to select features, applied the random forest algorithm to establish 7 classification models to distinguish between benign and malignant lung lesions, and used 1000 times 10-fold cross-validation to evaluate the diagnostic efficacy of the models. The established models were the CT feature model, ^18^F-FDG PET feature model, ^68^Ga-NOTA-PRGD2 PET feature model, CT & ^18^F-FDG PET feature model, CT & ^68^Ga-NOTA-PRGD2 PET feature model, ^18^F-FDG PET & ^68^Ga -NOTA-PRGD2 PET feature model, and the model of combination of all features. The results showed that the diagnostic efficacy of these seven models was good, and the corresponding average AUC values ​​were 0.870, 0.895, 0.853, 0.903, 0.876, 0.892, and 0.908. Considering the intuitive difference in AUC values, if these models must be sorted, we believe that the performance from high to low is as follows: the model of combination of all features, CT & ^18^F-FDG PET feature model, ^18^F-FDG PET feature model, ^18^F-FDG PET & ^68^Ga-NOTA-PRGD2 PET feature model, CT & ^68^Ga-NOTA-PRGD2 PET feature model, CT feature model, and ^68^Ga-NOTA-PRGD2 PET feature model. Previous studies (27) generally believed that CT & ^18^F-FDG PET feature models were better than ^18^F-FDG PET feature models, and the ^18^F-FDG PET feature model was better than the CT feature model. Our study also supported this result. In the future, different modeling methods could be used to try to develop a model with optimal prediction performance.

We also found that the AUC value of the three-modal image feature model was better than those of the dual-modal image feature models, and the AUC values of the dual-modal image feature models were usually better than those of the single-modal image feature models, which further extended the results of Zhang (27), Teramoto (30), and Blemker (31). This may result from the combination of multi-modality and multi-probe images extracting more lesion information than single-modality or single-probe image features and improving the diagnostic efficiency of the models. The multi-modality and multi-probe imaging method was certainly better than the single-modality or single-probe imaging method in the diagnosis of benign and malignant lung lesions, but in clinical practice, considering the high penetration rate, low cost and high sensitivity of CT, screening malignant lung lesions based on CT is still a good choice under limited conditions.

We discussed the radiomics model based on a new imaging method, ^68^Ga-NOTA-PRGD2 PET. The established model had an AUC value of 0.853, which was significantly lower than that of the ^18^F-FDG PET feature model (AUC of 0.895, P<0.001), and its sensitivity and specificity were both lower than the latter, which may indicate that the ^68^Ga-NOTA-PRGD2 PET feature model performed worse than the ^18^F-FDG PET feature model. The possible reason, on the one hand, was related to the modeling methods and deviation of VOI. On the other hand, the radiation dose of ^68^Ga-NOTA-PRGD2 PET was lower than that of ^18^F-FDG PET, which resulted in relatively poor imaging quality and less information. After the addition of CT, the diagnostic efficiency of both methods increased. The sensitivity of the CT & ^68^Ga-NOTA-PRGD2 PET feature model was even higher than that of the CT & ^18^F-FDG PET feature model, though with a lower AUC value (P<0.001). Besides, the addition of ^68^Ga-NOTA-PRGD2 PET features to the CT & ^18^F-FDG PET feature model can significantly improve the diagnostic efficiency of the model (AUC: 0.908 *vs*. 0.903, P = 0.003 < 0.05), which indicated the potential value of the multi-modality and multi-probe models and warranted further validation in the future. Our study did not report radiomics analysis on metastases detection of lung cancer based on ^68^Ga-NOTA-PRGD2 PET and it was our future direction.

Our study has some limitations as follows. (1) Our study was a retrospective analysis that enrolled 48 patients with difierent benign or malignant pulmonary space-occupying lesions. The amount of data was small, and all came from a single center, so we made efforts to eliminate the uncertainty and instability of the features as much as possible, such as using cross-validation instead of separating another validation set for the limited data and setting segmentation standards for extracting features from images. (2) In the differentiation of benign and malignant lesions, the number of benign and malignant cases was slightly unbalanced, which needs to be further considered in future studies.

In conclusion, this is a rather novel study to suggest that some ^68^Ga-NOTA-PRGD2 PET image features solely could have even better diagnostic capacity than all CT features for pulmonary space-occupying lesions. Apart from that, this is also a pilot study to show that classification models developed based on multi-modality and multi-probe images can extract more information about lesions and improve the diagnostic efficiency of radiomics models than single-modality and single-probe image models. For future study, our preliminary clinical hypothesis of using radiomics based on ^68^Ga-NOTA-PRGD2 PET images and multi-modality and multi-probe images as a diagnostic tool warrants further validation in a larger multicenter sample size.

## Data Availability Statement

The raw data supporting the conclusions of this article will be made available by the authors, without undue reservation.

## Ethics Statement

The studies involving human participants were reviewed and approved by Institute Review Board of Peking Union Medical College Hospital, Chinese Academy of Medical Sciences and Peking Union Medical College. Written informed consent for participation was not required for this study in accordance with the national legislation and the institutional requirements.

## Author Contributions

ZZ, FX, and KZ conceived of the project, analyzed the data, and wrote the paper. LL, LF, and XJ provided expert guidance, data, or analysis tools, and reviewed the manuscript. All authors contributed to the article and approved the submitted version.

## Funding

This study has received funding by the Capital Health Development Scientific Research Project (2018-1-4011), the Chinese Academy of Medical Science Clinical and Translational Medicine Research Foundation (2019XK320032), and the National Natural Science Foundation of China (81871392).

## Conflict of Interest

The authors declare that the research was conducted in the absence of any commercial or financial relationships that could be construed as a potential conflict of interest.

## Publisher’s Note

All claims expressed in this article are solely those of the authors and do not necessarily represent those of their affiliated organizations, or those of the publisher, the editors and the reviewers. Any product that may be evaluated in this article, or claim that may be made by its manufacturer, is not guaranteed or endorsed by the publisher.
