# Supplementary material for: A Pilot Study of Radiomics Models Combining Multi-Probe and Multi-Modality Images of 68Ga-NOTA-PRGD2 and 18F-FDG PET/CT for Differentiating Benign and Malignant Pulmonary Space-Occupying Lesions
Source: Front Oncol. 2022 Jun 2;12:877501. doi: 10.3389/fonc.2022.877501 (PMC9201288; doi:10.3389/fonc.2022.877501)
Supplement: Supplementary file 1 [file Table_1.docx]

Supplementary Material

# Supplementary Table 1 AUC values of features for predicting benign and malignant lung lesions and comparison of features between the benign and malignant groups

| Modality  Features | CT | | ^18^F-FDG PET | | ^68^Ga-NOTA-PRGD2 PET | |
| --- | --- | --- | --- | --- | --- | --- |
|  | AUC | P | AUC | P | AUC | P |
| HU/SUV_min_ | 0.606 | 0.279 | 0.566 | 0.505 | 0.516 | 0.879 |
| HU/SUV_mean_ | 0.542 | 0.681 | 0.657 | 0.108 | 0.604 | 0.289 |
| HU/SUV_std_ | 0.534 | 0.739 | 0.704 | 0.037** | 0.691 | 0.051* |
| HU/SUV_max_ | 0.660 | 0.103 | 0.718 | 0.026** | 0.653 | 0.119 |
| HU/SUV_Q1_ | 0.483 | 0.868 | 0.477 | 0.821 | 0.565 | 0.513 |
| HU/SUV_Q2_ | 0.502 | 0.990 | 0.562 | 0.528 | 0.609 | 0.268 |
| HU/SUV_Q3_ | 0.507 | 0.952 | 0.657 | 0.108 | 0.638 | 0.160 |
| SUV_peak sphere 0.5 mL_ | / | / | 0.714 | 0.028** | 0.631 | 0.182 |
| SUV_peak sphere 1 mL_ | / | / | 0.690 | 0.052* | 0.622 | 0.216 |
| TLG | / | / | 0.520 | 0.849 | 0.512 | 0.916 |
| HISTO Skewness | 0.603 | 0.295 | 0.567 | 0.502 | 0.581 | 0.411 |
| HISTO Kurtosis | 0.455 | 0.651 | 0.459 | 0.686 | 0.543 | 0.668 |
| HISTO ExcessKurtosis | 0.456 | 0.664 | 0.461 | 0.694 | 0.543 | 0.668 |
| HISTO Entropy log10 | 0.588 | 0.385 | 0.696 | 0.045** | 0.686 | 0.057* |
| HISTO Energy Uniformity | 0.574 | 0.453 | 0.648 | 0.131 | 0.688 | 0.055* |
| SHAPE Volume mL | 0.608 | 0.273 | 0.572 | 0.473 | 0.588 | 0.372 |
| SHAPE Sphericity | 0.529 | 0.775 | 0.678 | 0.068* | 0.767 | 0.006*** |
| SHAPE Compacity | 0.634 | 0.171 | 0.558 | 0.560 | 0.571 | 0.475 |
| GLCM Homogeneity Inverse difference | 0.495 | 0.971 | 0.677 | 0.070* | 0.788 | 0.003*** |
| GLCM Energy Angular second moment | 0.524 | 0.812 | 0.664 | 0.094* | 0.738 | 0.015** |
| GLCM Contrast Variance | 0.634 | 0.171 | 0.706 | 0.035** | 0.762 | 0.006*** |
| GLCM Correlation | 0.657 | 0.108 | 0.564 | 0.533 | 0.501 | 1.000 |
| GLCM Entropy log10 | 0.648 | 0.130 | 0.683 | 0.062* | 0.704 | 0.037** |
| GLCM Dissimilarity | 0.640 | 0.153 | 0.699 | 0.041** | 0.759 | 0.008*** |
| GLRLM SRE | 0.630 | 0.186 | 0.596 | 0.329 | 0.753 | 0.009*** |
| GLRLM LRE | 0.503 | 0.981 | 0.615 | 0.243 | 0.778 | 0.004*** |
| GLRLM LGRE | 0.558 | 0.559 | 0.495 | 0.972 | 0.597 | 0.323 |
| GLRLM HGRE | 0.576 | 0.439 | 0.683 | 0.062* | 0.622 | 0.216 |
| GLRLM SRLGE | 0.569 | 0.482 | 0.514 | 0.897 | 0.575 | 0.446 |
| GLRLM SRHGE | 0.586 | 0.384 | 0.697 | 0.044** | 0.637 | 0.166 |
| GLRLM LRLGE | 0.568 | 0.489 | 0.521 | 0.840 | 0.633 | 0.175 |
| GLRLM LRHGE | 0.551 | 0.608 | 0.626 | 0.199 | 0.543 | 0.668 |
| GLRLM GLNU | 0.582 | 0.404 | 0.634 | 0.173 | 0.648 | 0.131 |
| GLRLM RLNU | 0.630 | 0.186 | 0.486 | 0.896 | 0.491 | 0.934 |
| GLRLM RP | 0.630 | 0.186 | 0.588 | 0.372 | 0.741 | 0.013** |
| NGLDM Coarseness | 0.528 | 0.784 | 0.499 | 1.000 | 0.475 | 0.805 |
| NGLDM Contrast | 0.560 | 0.544 | 0.662 | 0.098* | 0.738 | 0.015** |
| NGLDM Busyness | 0.583 | 0.398 | 0.657 | 0.108 | 0.708 | 0.033** |
| GLZLM SZE | 0.616 | 0.238 | 0.707 | 0.034** | 0.595 | 0.340 |
| GLZLM LZE | 0.528 | 0.784 | 0.639 | 0.159 | 0.725 | 0.020** |
| GLZLM LGZE | 0.551 | 0.608 | 0.727 | 0.020** | 0.644 | 0.145 |
| GLZLM HGZE | 0.595 | 0.335 | 0.712 | 0.030 | 0.650 | 0.126 |
| GLZLM SZLGE | 0.573 | 0.460 | 0.794 | 0.003*** | 0.536 | 0.721 |
| GLZLM SZHGE | 0.608 | 0.273 | 0.713 | 0.028** | 0.653 | 0.119 |
| GLZLM LZLGE | 0.528 | 0.784 | 0.595 | 0.340 | 0.734 | 0.015** |
| GLZLM LZHGE | 0.531 | 0.757 | 0.655 | 0.113 | 0.678 | 0.069* |
| GLZLM GLNU | 0.612 | 0.253 | 0.532 | 0.748 | 0.475 | 0.803 |
| GLZLM ZLNU | 0.644 | 0.143 | 0.667 | 0.089* | 0.595 | 0.335 |
| GLZLM ZP | 0.641 | 0.150 | 0.655 | 0.113 | 0.729 | 0.019** |
| Note: *, 0.05≦P<0.1; **, 0.01≦P<0.05; ***, P<0.01, similarly hereinafter. | | | | | | |
